# Sensory translation between audition and vision

**DOI:** 10.3758/s13423-023-02343-w

**Published:** 2023-10-06

**Authors:** Charles Spence, Nicola Di Stefano

**Affiliations:** 1 Crossmodal Research Laboratory, University of Oxford, Oxford, UK; 2 Department of Experimental Psychology, New Radcliffe House, University of Oxford, Oxford, OX2 6BW UK; 3 Institute of Cognitive Sciences and Technologies, National Research Council of Italy (CNR), Rome, Italy

**Keywords:** Sensory translation, Colour music, Crossmodal correspondences, Perceptual similarity, Synaesthesia

## Abstract

Across the millennia, and across a range of disciplines, there has been a widespread desire to connect, or translate between, the senses in a manner that is meaningful, rather than arbitrary. Early examples were often inspired by the vivid, yet mostly idiosyncratic, crossmodal matches expressed by synaesthetes, often exploited for aesthetic purposes by writers, artists, and composers. A separate approach comes from those academic commentators who have attempted to translate between structurally similar dimensions of perceptual experience (such as pitch and colour). However, neither approach has succeeded in delivering consensually agreed crossmodal matches. As such, an alternative approach to sensory translation is needed. In this narrative historical review, focusing on the translation between audition and vision, we attempt to shed light on the topic by addressing the following three questions: (1) How is the topic of sensory translation related to synaesthesia, multisensory integration, and crossmodal associations? (2) Are there common processing mechanisms across the senses that can help to guarantee the success of sensory translation, or, rather, is mapping among the senses mediated by allegedly universal (e.g., amodal) stimulus dimensions? (3) Is the term ‘translation’ in the context of cross-sensory mappings used metaphorically or literally? Given the general mechanisms and concepts discussed throughout the review, the answers we come to regarding the nature of audio-visual translation are likely to apply to the translation between other perhaps less-frequently studied modality pairings as well.

## Sensory translation

The possibility of conveying the information associated with one sensory input by means of another or, at the very least, of trying to express one sensory impression by means of a sensation that is normally associated with the stimulation of another modality, has long attracted the interest of both scholars and artists. For example, a wide variety of inventors, designers, and artists have tried to translate music into light, colours and perfumes, while many novelists and poets have shaped their own style through the use of cross-sensory, or synaesthetic, metaphors, that is, ways of describing one kind of sensory experience by means of another presented in a different sensory domain. Here, exploiting the traditional meaning of translation, conceived as “a craft consisting in the attempt to replace a written message and/or statement in one language by the same message and/or statement in another language” (Newmark, 1981, p. 7; see also Aguiar & Queiroz, 2013), we propose to refer to this broad spectrum of phenomena with the umbrella term ‘sensory translation’ (see Table 1 for a definition of a number of the key concepts appearing in this review).

**Table 1:** Definition of key concepts appearing in this review

Key Concept	Definition	Relevant Sources
Translation	The attempt to replace a written message and/or statement in one language by the same message and/or statement in another language	Newmark, 1981
Sensory translation	The attempt to convey the meaning associated with one sensory stimulus by means of a stimulus presented in a different sensory modality	This review
Crossmodal correspondences	Deliberate and consistent matching between perceptual stimuli, attributes, or dimensions from different sensory domains that are observed in normal perceivers (i.e., non-synaestheses)	Spence, 2011
Synaesthesia	Neurological condition in which specific inducing stimuli give rise to an additional concurrent experience in either the same or different sensory modality	Grossenbacher & Lovelace, 2001
Inducer	The stimulus that triggers the synaesthetic perception	Grossenbacher & Lovelace, 2001
Concurrent	The stimulus that is elicited by the perception of the inducer in synaesthesia	Grossenbacher & Lovelace, 2001
Synaesthetic metaphors	Linguistic metaphors that cross the senses (e.g., ‘dark melodies’)	Hunt, 2005; Marks, 1996; Shen & Gil, 2007; Williams, 1976; Winter, 2011
Amodal dimensions	Properties of perceived objects and events that are allegedly common across different senses	Bahrick, 2009; Marks, 1978
Prothetic dimensions	Quantitative perceptual continua that have a clear ‘more than’ and ‘less than’ end	Stevens, 1957
Methatetic dimensions	Perceptual dimensions that obey a structured organization without necessarily having a ‘more than’ or ‘less than’ end	Stevens, 1957
Unidirectional/bidirectional	Referred to perceptual or sensory phenomena that occur in only one privileged direction, i.e., from one sense to another (unidirectional), or else in both directions (bidirectional).	Gil & Shen, 2021; Zhou & Tse, 2022

The idea of the possibility of sensory translation might sometimes be rooted in the speculative historical claim that, although the senses can be conceived of as different channels with which to gather information from the environment, they must share some general functioning mechanism as they are all expressions of the same human perceptual abilities (e.g., Aristotle, 1907; see also Marks, 1978). Indeed, go back to ancient alchemical texts, and one sometimes comes across tables explicitly linking specific sounds to particular colours (e.g., see Huidobro Moya, 2007). Interestingly, scholars have suggested that the ancient Maya and Mesoamerican peoples experienced the senses—at least smell, hearing and sight—“as linked in a near-synaesthetic fashion” (Houston & Toube, 2000, p. 261). In Western philosophy, early conceptualizations of the senses stressed the distinction between different modalities while, at the same time, also emphasizing the intimate link between them. For instance, Aristotle presented the *sensus communis* as a putative psychological function connecting sensory impressions gathered from the five senses and processing them as a whole (Aristotle, 1907; see Johnstone, 2021).

In recent years, the question of whether it is possible to meaningfully connect the senses (or, at the very least, a subset of sensory impressions) has taken on renewed interest amongst those scientists trying to develop more intuitive sensory substitution devices (to compensate for sensory loss, e.g., in blind or deaf people, see Abboud et al., 2014; Hamilton-Fletcher et al., 2016; Marks, 1983; Spence, 2018, as well as for [sensory] augmentation, e.g., Akinbiyi et al., 2006; Quek et al., 2015; Pinardi et al., 2023). Sensory translation also appears frequently in texts on semiotics (e.g., Gottlieb, 2018), and has been gaining traction within the literature on museum studies as well (e.g., Liao, 2018; Neather, 2008, 2012; see also Cecon, 2011).

Over the centuries, a number of inventors and artists have been interested in trying to translate music into harmonious light displays/concerts, as with Alexander Wallace Rimington’s colour organ (Jewanski, 2010a; Moritz, 1997; Peacock, 1988; Plummer, 1915; Rimington, 1895; Schöffer, 1985; Sullivan, 1914; see also Bragdon, 1916, 1918; Hector, 1922).^1^ Cornelius Drebbel (1572–1633) is known as the inventor of musical instruments, such as virginals or lutes that would automatically translate light into sound (Gouk, 2000; Wilkins, 1680). However, the precise functioning of these innovative devices is not entirely clear. According to Wilkins (1680, pp. 148-149) when the instrument was placed in the sun, it would start to emit harmonies that were pleasant and soft. When moved into the shade it would become silent. This effect was allegedly caused by the warmth of the sun which affected some moisture in the instrument and the density on the air in it thus making its strings vibrate. The existence of this and similar instruments demonstrate that the human mind has long been attracted by exploiting the allegedly natural connection between sound and light and hence the possibility of translating something of the meaning or feeling associated with one sensory input into a stimulus from a different sensory modality (see also Mather, 2015).^2^

Other creative individuals, meanwhile, have wanted to convert music into perfumed performances (e.g., see Piesse’s, 1867, 1891, ‘Gamut of Odors’; and see Spence, 2021, for a review), or to deliver taste concerts by means of specially-constructed flavour organs (e.g., see ‘The taste organ’, 1926).^3^ Amongst the recent attempts to translate between audition and olfaction, Chang Hee Lee’s (2013) project “Essence in Space” is worth mentioning.^4^ In this case, the translation is fixed by an adapted keyboard in which each key is mechanically linked to a fragrance situated below the keyboard. As each key is pressed, a droplet of perfume is released and collected in a bottle. This process continues as each key is struck, resulting in a mixture of different perfume droplets being collected. At the end of the ‘performance’, a unique blend of perfume has been created based on the olfactory conversion of musical “ingredients”. In all such cases, those involved would appear to believe that there was a possibility of meaningfully connecting one sensory impression to another. Rarely, if ever, does one come across people proposing arbitrary cross-sensory mappings (i.e., under the assumption that any translation would be as good, or bad, as another). In a pedagogical context, Nijs et al. (2012) developed the Music Paint Machine, an interactive music system that translates movement and sound into colours, allowing a musician to create digital painting by playing an acoustic musical instrument and by moving on a coloured pressure mat. The system was conceived as a learning tool mainly aimed at the development of musical creativity and at strengthening the relationship between the musician and their instrument.

Around the start of the 20^th^ century, many writers, including novelists and poets such as Charles Baudelaire (Anderson, 1980; Baudelaire, 1857, 1954) and Emily Dickinson, experimented with synaesthetic, or cross-sensory, metaphors in their work (e.g., Gibson, 1969; Harrison, 2001; von Erhardt-Siebold, 1932). In such cases, the artist’s intuitive attempts to express one kind of sensory experience by means of another were often based on their own synaesthetic experiences (e.g., Di Stefano et al., 2022a; Marks, 1978). Intriguingly, however, such synaesthetic metaphors are typically unidirectional^5^ (Shen & Cohen, 1988; cf. Zhou & Tse, 2022), just as for synaesthesia proper (Deroy & Spence, 2013).

Over the last quarter of a century or so, a growing number of marketers and advertiser have become increasingly interested in the question of whether they can communicate (more) effectively with their customers by means of synaesthetic marketing (e.g., see Bolognesi & Strik Lievers, 2018; Crisinel & Spence, 2012a; Dunne, 2014; Kiefer, 2017; Meehan et al., 1998; Nelson & Hitchon, 1995, 1999; Spence, 2012). The latter can presumably be considered as yet another kind of sensory translation. Similarly, contemporary wine writers have been known to resort to a range of synaesthetic metaphors when trying to express the experience of flavour by means of musical analogies (Caballero, 2009).^6^ Intriguingly, if somewhat obscurely, Paradis and Eeg-Olofsson (2013, p. 22) have suggested that when terms such as ‘sharp’, ‘soft’, ‘lemon’, and ‘cherry’ are used to describe a wine’s perceptual qualities, they should not be considered to be “polysemous synesthetic metaphors, but monosemous synesthetic metonymizations, more precisely zone activations.” In this passage, it would seem that the authors would like to point out that the perception of such crossmodal similarities suggests that transitions across sensory domains in human language and understanding are not idiosyncratic, and tend to maintain the original semantic meaning unaltered (i.e., it is monosemous) rather than extending the literal meaning metaphorically to other domains (see also Rakova, 2003). Regardless, these phenomena fit with a growing variety of multisensory experiences, such as tasting events in which wine and music are deliberately paired (see Spence & Wang, 2015a, b, c, for reviews; and Spence, 2019a, for a recent review). Indeed, the topic of synaesthetic design has become increasingly popular in recent years (Haverkamp, 2014).

As will become clear, the concepts of synaesthesia, synaesthetic metaphor, and crossmodal associations play a key role throughout this review. While these concepts are closely linked, and have been often wrongly conceived as referring to the same phenomenon (i.e., as synonyms), they are also importantly different (as stressed by Cazeaux, 2002).^7^ Synaesthesia refers to a rare neurological condition in which specific inducing stimuli give rise to an additional idiosyncratic concurrent experience in either the same or different sensory modality (Grossenbacher & Lovelace, 2001). Several different theories have been put forward to explain the existence of synaesthesia (see Simner & Hubbard, 2013, for a review). Here, we will be particularly interested in those cases of synaesthesia in which an inducer in one sensory modality gives rise to a concurrent in a different sensory modality, such as when hearing musical sounds elicits coloured concurrents (e.g., MacDougal, 1898).

The term ‘synaesthetic metaphor’ is typically used to refer to those linguistic metaphors that cross the senses, in expressions such as ‘a sharp-tasting cheese’, or ‘he is fond of wearing loud red trousers’. Such cross-sensory metaphor has been labelled synaesthetic because the cross-sensory expressions are similar, at least according to certain commentators, to the unusual inducer-concurrent mappings experienced by those synaesthetes who experience crossmodal forms of the condition. Some researchers have labelled such expressions as a kind of verbal synaesthesia (e.g., Popova, 2005). Finally, crossmodal associations, often referred to as crossmodal correspondences (Spence, 2011), are also often surprising to people when first hearing about them, just like synaesthesia. Crossmodal correspondences have been defined as the tendency for a sensory feature, attribute, or dimension in one sensory modality, either physically present, or merely imagined, to be matched (or associated) with a sensory feature, attribute, or dimension in another modality (Spence, 2011). Unlike synaesthesia, which is, by definition, idiosyncratic in terms of the inducer-concurrent mapping (Deroy & Spence, 2013; Grossenbacher & Lovelace, 2001), crossmodal correspondences tend to be consensual (see also Sun et al., 2018). The majority of people will, for example, associate round shapes with sweetness, and angular shapes with sour-tasting foods (Spence & Deroy, 2012). Furthermore, unlike is so often the case for synaesthetic metaphors (which typically only work, or really make sense, in one direction; see Chan et al., 2013; Gil & Shen, 2021; Zhou & Tse, 2022), the correspondences operate bidirectionally in the majority of cases (see Deroy & Spence, 2013; see also Smith, 1987, p. 94). So, while it makes sense to call cheese ‘sharp’, describing sharpness as a cheese really doesn’t work. By contrast, people are as happy to agree that sweet tastes are round (i.e., rather than sharp) as they are to match roundness with sweetness.

However, complicating matters somewhat, synaesthetes experience crossmodal correspondences (in this, they are no different from non-synaesthetes). On occasion, the inducer-concurrent mapping experienced by the synaesthete may coincide with the crossmodal correspondences experienced by the population at large. Intriguingly, while both synaesthesia and synaesthetic metaphor are typically considered to be unidirectional phenomena, the crossmodal correspondences that have been documented to date are mostly bidirectional, thus highlighting a potentially important distinction between these superficially similar empirical phenomena (see also Deroy & Spence, 2013, for a summary of similarities and differences between crossmodal correspondences and synaesthesia).

When the diverse literatures are taken together, it soon becomes clear how widespread the interest in sensory mapping/translation really is (cf. Misdariis et al., 2021; Trotta et al., 2020, for a couple of other recent examples). Beyond its relevance to artistic performance or marketing/design contexts, we suggest that such a widespread interest might be rooted in the nature of the topic itself, which raises a number of intriguing (not to mention challenging) issues (see Daniels et al., 2010; Daniels & Naumann 2015). In this narrative historical review (see Ferrari, 2015; Furley & Goldschmied, 2021, on the strengths of narrative-style reviews), we attempt to shed some light on the topic by answering the following questions: (1) How is the topic of sensory translation related to synaesthesia, multisensory integration, and crossmodal associations? (see “Synaesthetic Translations”); (2) Are there common processing mechanisms across the senses that guarantee the success of sensory translation or, rather, is the mapping between the senses mediated by allegedly universal (e.g., amodal) stimulus dimensions? Answering these questions (see “Putative Mechanisms Underlying Sensory Translation” and “Problems for Any Attempt to Translate Directly, or ‘Literally, Between the Senses”), in turn, allows us to provide an answer to the following, more general, question as well: (3) Is the term ‘translation’ in the context of cross-sensory mappings used metaphorically or literally? The hope is that addressing these questions will help us to understand why it is that sensory translation has been considered, across the millennia, as a source of literary or poetic inspiration, a matter of philosophical reflection, and a research question worthy of serious empirical investigation.

The primary focus of the remainder of this review will be on the translation between audition and vision. In part, this simply reflects the fact that there is far more research on this pair of sensory modalities than for any other and, in part, the narrowing of focus was needed in order to prevent the review from becoming overly long. Nevertheless, the answers we come to regarding the nature of sensory translation between this frequently-studied pair of sensory modalities likely extend to the translation between other perhaps less frequently studied/mentioned modality pairings as well.

## Synaesthetic translations

### Synaesthetic perceptions

Historically, the most common source of inspiration for those wanting to express auditory(/visual) sensations by means of visual(/auditory) ones has been the vivid, yet typically idiosyncratic, concurrents experienced by those individuals with synaesthesia. In fact, ‘coloured hearing’ turns out to be one of the most commonly-mentioned forms of synaesthesia, and often appeared in the scientific literature in the decades around 1900 (e.g., Argelander, 1927; Dauriac, 1902; de Parville, 1883; English, 1923; Flournoy, 1893; Ginsberg, 1923; Jewanski et al., 2009, 2011, 2020; Suarez de Mendoza, 1890; Underwood, 1893; Zigler, 1930). Both pitch (defined as a perceptual property of sounds that allows for their ordering on a frequency-related scale; see Zwicker & Fastl, 2013, p. 111) and timbre (also known as tone colour, or tone quality, from psychoacoustics, refers to the perceived sound quality of a musical note, sound or tone, see McAdams, 2019) appear to be salient auditory features (i.e., sensory inducers) driving the various coloured musical concurrents that have been reported in the literature (e.g., Curwen, 2018; Itoh et al., 2017; Marks, 1975). At the same time, however, it has also been acknowledged that there may be a strong visual mental imagery component to many coloured responses to music (Ahsen, 1997; Karwoski et al., 1942; Mudge, 1920; see also Mills et al., 2003; Nanay, 2018, 2020; Spence & Deroy, 2013). Referring to the evoked sensory experience in such cases in terms of ‘mental imagery’, rather than describing it as a synaesthetic concurrent, helps to draw attention to the fact that the qualities of the latter, such as the evoked colour coming or going, or else fading during a musical performance (see MacDougal, 1898; Riggs & Karwoski, 1934; Underwood, 1893), are not typical of synaesthesia as it tends to be conceptualized nowadays (see Spence & Deroy, 2013; though see also Nanay, 2018, 2020).

Many of the artists and composers who were interested in ‘colour music’ (e.g., Klein Cornwall-Clyne, 1937; Zilczer, 1987; see also Alves, 2005; Galeyev, 1976, 2003; McKellar, 1972, 1997; and Spence & Di Stefano, 2022b, for a recent review), such as, for example, Kandinsky (1977) and Scriabin, purportedly based their works on, or at the very least were inspired by, their own synaesthesia (e.g., Denham, 2017; Galeyev & Vanechkina, 2001; Harrison, 2001; Ione & Tyler, 2003, 2004; Kandinsky, 1977; Myers, 1911, 1914; Peacock, 1985; Witztum & Lerner, 2016; see Spence, 2020b, for a review). For example, Kandinsky (1977) suggested that the sound of the trumpet is scarlet (see Ione & Tyler, 2003, 2004; Just, 2017; though see also O’Regan, 2011). Kandinsky (1977) referred to a number of specific colour–sound mappings in his writings. However, it is often unclear whether the examples provided were based on the artist’s own synaesthesia, or else are perhaps better considered as examples of emotionally-mediated crossmodal correspondences (and hence might perhaps be expected to be experienced by us all; see Spence, 2020a, for a review), thus raising issues concerning how individual differences might affect the translation between the senses.

Something of a similar challenge faces those interested in trying to understand more about the idiosyncratic crossmodal mappings that have been suggested by synaesthetic Russian artists—namely, the composers Rimsky-Korsakov (who reported ‘seeing’ music in the key of A-major as yellow; Myers, 1911), and Scriabin (Galeyev & Vanechkina, 2001; Myers, 1914). Once again, though, it has long been the subject of debate as to what exactly the relationship, if any, was between Scriabin’s personal repertoire of idiosyncratic audiovisual inducer-concurrent mappings, and those chosen for his colour circle/score/luce (see Galeyev & Vanechkina, 2001; Triarhou, 2016). At this point, it is worth stressing that there is no real “translation” between two perceptual (and actually perceived) stimuli in the case of synaesthesia. More properly, with respect to the specific stimuli that are being linked (or associated), synaesthesia seems to be more related to synonymity, or identity, rather than necessarily translation since, for a synaesthete, the sound of the trumpet and the colour scarlet are simply part of one and the *same* perceptual experience (that is, the inducer is always co-experienced with the concurrent independent of the actual desire, or intention, of the perceiver).

Going deeper into the concept of translation, synaesthesia can perhaps be considered as a case of literal translation (Newmark, 1981), albeit one that is legitimated by “private” rules. That is, given the idiosyncratic nature of the synaesthete’s inducer-concurrent mappings, the concurrent can be seen as a faithful translation of, or synonymous with, the inducer, but it is faithful only for the individual synaesthete. By contrast, sensory translation can be considered more as a translation based on idiomatic and allegedly more universal criteria which can be shared across, or accessed by, normal perceivers. This means that synaesthetes cannot experience, for instance, the sound of the trumpet without, at the same time, also being aware of the colour scarlet, just as an English speaker cannot conceive of the terms ‘happy’ and ‘joyful’ as being unrelated. By contrast, nonsynaesthethes can experience roundness as unrelated to sweetness, just as an English speaker can conceive the term happy without necessarily thinking at the Italian translation “felice”.

Moreover, while it has been suggested that the inducer-concurrent mappings experienced by synaesthetes typically tend to be appreciated by non-synaesthetes (e.g., see Ward et al., 2008), it is important to note that this has no necessary implications for the question of whether the inducer can be (even metaphorically) considered as a ‘translation’ of the concurrent in the case of synaesthesia. According to O’Malley (1957, p. 393), synaesthesia might imply some loss of perceptual differentiation and discrimination between the inducer and concurrent, thus ruling out the possibility of looking for, or establishing, a comparison criterion underlying the association. As such, and especially because of the idiosyncratic nature of the inducer-concurrent mapping, many researchers have been driven to search for alternative approaches to sensory translation that do not rely on the synaesthete’s own idiosyncratic mappings from one sense to another. In the next section (“Putative Mechanisms Underlying Sensory Translation”), we examine whether the idea that the structural relationships between stimulus dimensions can help to provide a more robust means of translating between the senses, at least for a subset of stimulus dimensions.

### Synaesthetic metaphors

Synaesthetic metaphors are expressions that “transfer one sense to another” (De Ullmann, 1945, p. 813; see also Shen, 1997) by establishing a relation between elements that are apparently semantically incompatible. For example, saying that a melody is sweet implies attributing a quality of taste to sounds, which do not manifest themselves through taste attributes. When it comes to the use of synaesthetic metaphors, there is an initial question as to which comparison modality people will intuitively gravitate toward and, thereafter, or perhaps as part of one and the same decision, which dimensions of sensory experience they deem it most appropriate to compare (Cazeaux, 2002; Motoki et al., 2020; Motoki & Velasco, 2021). Put more concretely, if one were to try and express the sound of the trumpet crossmodally, people might spontaneously reach for a visual metaphor (such as the colour scarlet) or perhaps a taste descriptor, an aroma, or perhaps a tactile texture instead (Shibuya et al., 2007). Over the years, a number of researchers have analyzed the patterns of ‘synaesthetic metaphor’ in both literature and poetry (see Hunt, 2005; Marks, 1996; Shen & Gil, 2007; Williams, 1976). Their findings provide insights concerning the modalities of expression (in terms of the direction) that occur most frequently in literary texts (see also Fishman, 2022).^8^

**Fig. 1:**
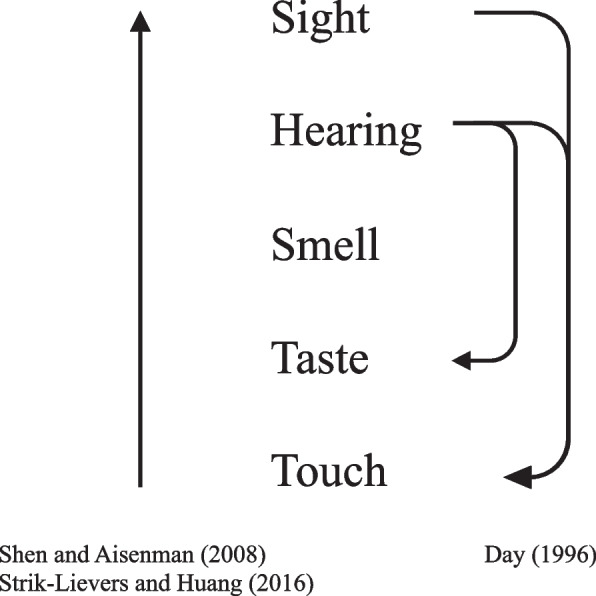
Hierarchical organization of the senses based on their purported contribution to conceptualization and mental activity, ranking from the ‘highest’ modality—sight—to the ‘lowest’ sense—namely, touch (e.g., see Cytowic, 1989a, b, 1993; Day, 1996; Müller et al., 2022; Tsur, 1992; Ullman, 1957; Williams, 1976). Arrows indicate the most common direction of the occurrence of synaesthetic metaphors that are found in literary sources (Day, 1996), nonliterary texts (Strik-Lievers & Huang, 2016), and in both sources (Shen and Aisenman, 2008). [Reproduced from Di Stefano et al., 2022a]

Many scholars (e.g., see Cytowic, 1989a, b, 1993; Day, 1996; Tsur, 1992; Ullman, 1957) have suggested that the perceptual modalities are organized along a scale ranging from the ‘highest’ modality—sight—followed by sound, smell (olfaction), taste (or gustation)—through to the ‘lowest’ sense—namely, touch (see also Houston & Taube, 2000, for a similar hierarchy in ancient non-Western populations). According to Shen and Aisenman (2008), who reviewed a large corpus of literary and non-literary sources from different languages and cultures, synaesthetic metaphor (sometimes referred to as poetic metaphor, Marks, 1982a, b; linguistic synaesthesia, O’Malley, 1957; Sliz, 1942; or multisensory metaphor, Forceville, 2006) exhibit a robust, universal, tendency to use the ‘lower-to-higher’ structure more frequently than the inverse one (see Fig. 1). According to Conceptual Metaphor Theory (Lakoff & Johnson, 1980; see Landau et al., 2010, for a review), concrete terms provide the scaffold for more abstract terms/concepts. One might thus wonder if this concrete to abstract continuum can simply be extended to the case of synaesthetic metaphors (though see Paradis & Eeg-Olofsson, 2013, for an alternative viewpoint).^9^

Many scholars have, though, wanted to argue against the existence of such a hierarchy amongst the senses. Perception is by its very nature multisensory (e.g., Spence & Bayne, 2015), typically involving the simultaneous stimulation of several senses with different sources of sensory information potentially overlapping. The encoding of such multisensoriality in language might also affect the quality of information that is conveyed or else the exclusion of certain aspects of the overall sensory experience (Winter, 2019). In this regard, language may represent something of a “biased” field in which to investigate sensory perception, as the mediation of cognitive structures might serve to reduce the complexity of sensory perception (see also Fishman, 2022; Gil & Shen, 2021; Pedović & Stosić, 2018; Simner et al., 2010). Combining neuropsychological evidence with the literature from the fields of cognitive and perceptual psychology, Winter (2019) argued against the very idea of synaesthetic metaphors, suggesting that the latter are “neither synaesthetic nor metaphorical”, rather reflecting the way language and perception are related and how sensory content is encoded in the lexicon of human languages.

Moreover, if metaphor can be used to explain certain associations, e.g., those between vision and audition, it may be somewhat harder to do for other sensory domains, such as olfaction, due to the apparent difficulty of establishing the underlying basis for the metaphor. For example, the results of a crossmodal matching task reported by Belkin et al. (1997) revealed the existence of a correlation (or correspondence) between auditory pitch and a range of olfactory stimuli. However, the exact nature of the underlying feature(s) responsible for this crossmodal alignment remains unclear. The authors hypothesized that participants might have based their matches on olfactory dimensions expressed in semantic terms, such as dull-aromatic (Wender, 1968), heavy-light, bright-dark, or hard-soft (Klutky, 1990).^10^ At the same time, however, Belkin et al. also note that if these associations were to have been based on metaphors then the latter were, at the very least, elusive both to the participants and to the experimenters who were studying them (see also Dubois, 2007; Juhasz, 1926, cited in Hartshorne, 1934, p. 240; Pomp et al., 2018).

## Putative mechanisms underlying sensory translation

### Structural mapping

Sir Ernst Gombrich (1960), the famous art historian, once suggested that we should focus our attention on the structural relationships within the sensory systems rather than focusing on the similarity of specific elements when considering the nature of crossmodal associations. He suggested that “the problem of synesthetic equivalences will cease to look embarrassingly arbitrary and subjective if we fix our attention not on likeness of elements but on structural relationships within a scale or matrix” (p. 314). Gombrich was seemingly referring to the way in which stimulus dimensions are organized within each sensory modality. In this respect, it might be worth going back to Stevens’ (1957) early distinction between ‘metathetic’ and ‘prothetic’ sensory dimensions, with prothetic dimensions consisting of quantitative perceptual continua that have a clear ‘more than’ and ‘less than’ end. Examples of such prothetic sensory dimensions include loudness, brightness, lightness, heaviness, duration, and roughness. Metathetic dimensions, by contrast, tend to obey a well-structured organization without necessarily having a ‘more than’ or ‘less than’ end (see Table 1). Stevens classified the latter perceptual continua as ‘what kind’ or ‘where (position)’. For example, pitch is mentioned as a metathetic stimulus dimension, since a high-pitched tone is different in kind from a low-pitched tone, without necessarily being meaningfully related in a more than/less than way. Given this distinction, it seems reasonable to consider whether it is possible to translate between different prothetic dimensions by figuring out, or assuming, the relative position of the stimuli along their respective unisensory dimensions (cf. Cohen, 1934; Mellers & Birnbaum, 1982; Moul, 1930; Simpson et al., 1956, p. 100).

**Fig. 2:**
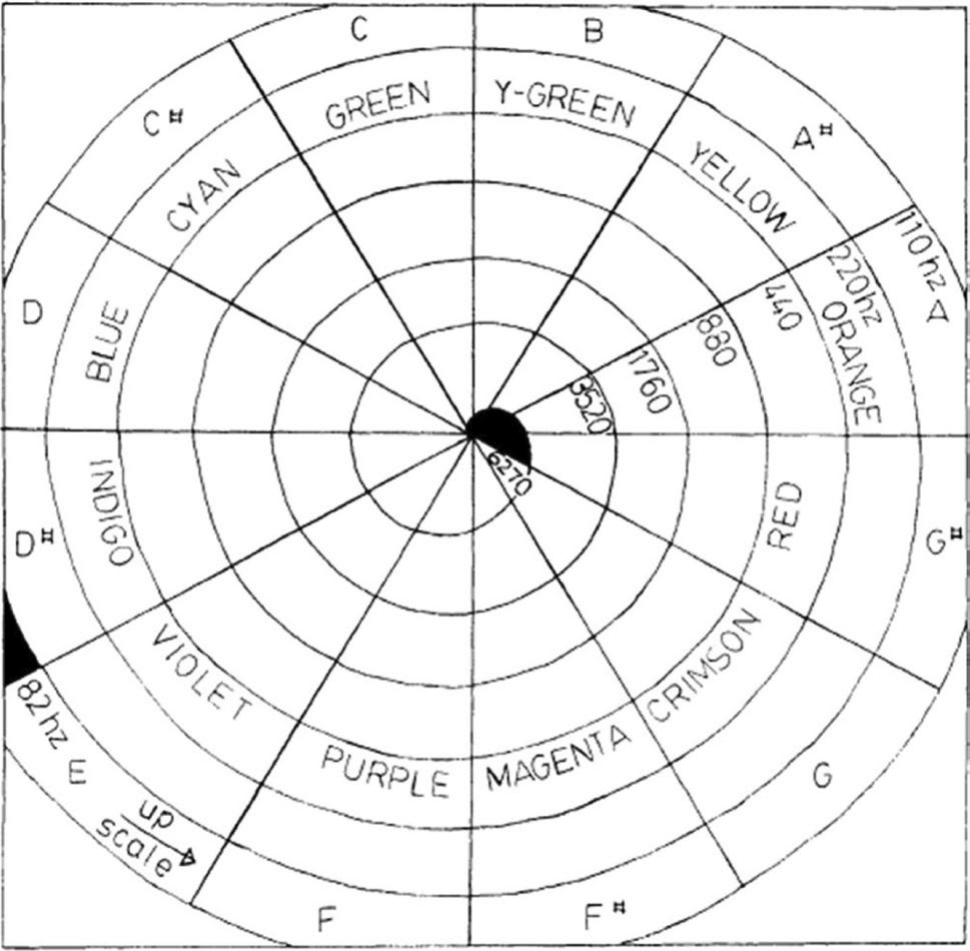
Layout of Pridmore’s (1984) final display panel. Notice how a given tone (e.g., C#) in all octaves (a musical octave is defined as a series of eight notes occupying the interval between [and including] two notes, the lower having half the frequency of vibration of the higher) is represented by a constant hue (e.g., cyan). Each octave is represented by a cycle, and each semitone (and its hue) by a sector (as presented in Pridmore, 1992)

It might not be so surprising, therefore, that many of those who have attempted to translate between the senses (including artists, scientists, and writers) have chosen to match hue with pitch (Jewanski, 2010a; Sabaneev & Pring, 1929; Sabaneyev, 1911). This choice of modalities/dimensions may, in part, be related to the fact that we are all predominantly visual creatures (Hutmacher, 2019; see also Winter et al., 2018), and that, in vision, colours are particularly salient features of perceived objects. This, along with the fact that the structure of both auditory pitch and colour space can be represented metathetically (see Stevens, 1957) would also seem to have provided sufficient grounds for many to want to figure out some kind of meaningful relationship between this particular pair of sensory dimensions (see Spence & Di Stefano, 2022b, for a recent review). Importantly, according to Pridmore (1992), hue and pitch are also the only circular perceptual dimensions thus providing another reason for wanting to connect them (see Fig. 2).

Over the centuries, many different authorities have put forward their own solutions as to what might be the most appropriate translation between pitch and hue (e.g., see Field, 1835; Galeyev & Vanechkina, 2001; Goethe, 1810/1840, c. 201–202, para. 748; Lavignac, 1899; Newton, 1704; see Table 2). This trend has continued over the last century, with a wide range of authorities, from architects to music scholars/teachers, and from psychologists to inventors, all putting forward their own (as it happens, somewhat idiosyncratic) cross-sensory mappings (e.g., Caivano, 1994; Davis, 1979; Galeyev & Vanechkina, 2001; Garner, 1978; Pridmore, 1992; Sebba, 1991; Wells, 1980). However, whenever experimental psychologists have attempted to demonstrate the influence of such crossmodal correspondences (e.g., between hue and pitch), using, for example, the speeded classification task, they have not been especially successful (see Bernstein et al., 1971; Melara, 1989), or else their results have been queried by psychologists on methodological grounds. So, for example, one of the main criticisms that has been levelled at Simpson et al.’s (1956) classic study, apparently showing a relationship between pitch and hue, is that the lightness of the visual stimuli may not have been controlled and hence any crossmodal mapping that was reported may actually reflect a lightness-pitch correspondence instead (e.g., Wicker, 1968).

**Table 2:** Correlation of colour and notes of the chromatic scale. Table highlighting the various crossmodal correspondences that have been proposed since Newton (1704). Newton’s correlation conforms to the seven-tone scale which he was probably familiar with. Castel’s correlations were made with the 12-tone chromatic scale, but as Wells (1980) notes, the hues fall in frequency as the tones rise in frequency. The alignment is reversed for the following correlations. The scale attributed to E. G. Lind presents the pitch of tones (sound frequency, Hz) and the frequency of light (presented in parentheses in the Table as Terahertz (THz), for example red is 476 THz). Rimington’s (1895) crossmodal mapping from Peacock (1988). Adapted from Wells (1980, Table 1)

Note	Newton	Castel	Finn	Rimington	Lind	Maryon
	1700	1720-1735	1881	1893	1900	c. 1920
C	Red	Blue	Red	Deep red	259 Hz, red (476)	Red
C#		Sea green, blue-green	Vermillion	Crimson		Red-orange
D	Orange	Green, bright green	Orange	Orange-crimson	289 Hz, orange (511)	Orange
D#		Olive, yellow-green	Yellow			Orange-yellow
E-flat				Orange		
E	Yellow	Yellow	Yellow-green	Yellow	322 Hz, yellow (546)	Yellow
F	Green	Apricot, yellow-	Green	Yellow-green	342 Hz, green (588)	Yellow-green
		orange, aurora				
F#		Orange	Blue-green	Green		Green
G	Blue	Red	Turquiose blue	Bluish green	385 Hz, blue (630)	Blue-green
G#		Crimson	Blue			Blue
A-flat				Blue-green		
A	Indigo	Violet	Indigo	Indigo	427 Hz, indigo (665)	Blue-violet
A#		Agate, blue-violet,	Violet			Violet
		light purple				
B-flat				Deep blue		
B	Violet	Indigo	Purple	Violet	485 Hz, violet (721)	Violet-red

In summary, given the fact that neither synaesthesia (see Synaesthetic Translations), nor the structural approach to capturing, or establishing, perceptually meaningful audiovisual associations works, it would seem appropriate to look for an alternative solution, or theoretical framework, with which to conceptualize the crossmodal translation/matching of pitch and hue.

### Perceptual similarity

As O’Malley (1957) observed, the “metaphorical fusion of different sense data must always have carried intimations of a plane or phase of reality in which there was indeed a sharing, an interchange of properties” (p. 409), thus suggesting that sensory translation might well be rooted in those properties that are shared by different stimuli. Indeed, the existence of consistent crossmodal associations might be taken to reveal that the stimuli that are paired are, in some respect at least, similar (O’Malley, 1957, p. 392). Going back to the audiovisual domain, while the various structural attempts to match pitch with hue have seemingly failed to arrive at any consensus, it is noticeable how, when freed from the constraint of having to align dimensions of sensory experience due to their structural similarity, some authors have instead reached for timbre-hue crossmodal mappings instead. In fact, long before Kandinsky came out with his suggestions concerning the crossmodal association between the sound of a trumpet and the colour scarlet (see Kandinsky, 1977), both Locke (1690) and Leibniz (1704/1896) had already put forward essentially the same crossmodal mapping when considering the experience of a blind man. The composer Raff also reported that he perceived the colour of the sound of the trumpet to be scarlet (other people apparently report it to be bright red; Ortmann, 1933), while, for Kandinsky, the sound of the tuba was also red (see also Anikin & Johansson, 2019; Donnell-Kotrozo, 1978; Ginsberg, 1923, p. 589; Menouti et al., 2015; Reuter et al., 2018b). Other contemporary researchers, meanwhile, have chosen to study the correspondences between timbre and shape (see Adeli et al., 2014; Gurman et al., 2021) or harmony (namely, consonance and dissonance) and visual roughness (Giannos et al., 2021; see Di Stefano & Spence, 2022, for a review on multisensory roughness, and Di Stefano et al., 2022b for a review on consonance and dissonance) (Table 3).

**Table 3:** Summary of different kinds of audiovisual crossmodal mappings/translations based on different kinds of stimulus dimension

	Visual dimension		
Auditory dimension	Brightness (Prothetic)	Hue (Circular & Metathetic)	Shape (No obvious organization)
Loudness (Prothetic)	Putatively amodal (e.g., Lewkowicz & Turkewitz, 1980; Walker-Andrews, 1994), and hence also structurally-based) Loudness-luminosity (Caivano, 1994); Can also be considered as prothetic correspondence (Spence et al., 2013); NB. Lightness & brightness may elicit different correspondences; (Marks et al., 1987)	Anikin & Johansson (2019) report loudness associated with saturation; Hamilton-Fletcher et al. (2017) correspondence between frequency & chroma; (NB. Colour sometimes confounded with lightness)	No evidence
Pitch (Circular & Metathetic)	See Wicker (1968); Polarity mapping/ correspondence between low vs. high pitch; white vs. black. (Ludwig et al., 2011; Marks, 1987a; Melara, 1989); (NB. in polar correspondences, it tends to be the relative, rather than absolute pitch that matters, see Spence, 2019b)	Structural alignment of circular dimensions (Caivano, 1994; Field, 1835; Galeyev & Vanechkina, 2001; Goethe, 1840; Lavignac, 1899; Newton, 1704; Pridmore, 1992; Sebba, 1991; Wells, 1980). (NB. Pitch change may introduce loudness difference)	Turned into polar mapping between low vs. high pitch, and round vs. angular shape (Marks, 1987a; Parise & Spence, 2012; Wellek, 1927)
Timbre (No obvious organizational principle)	No evidence	Anikin & Johansson (2019); Donnell-Kotrozo (1978); Menouti et al. (2015); Mudge (1920); Reuter et al. (2018a)	Adeli et al. (2014); Gurman et al. (2021); (NB. Smith & Sera, 1992, suggest that shape is a metathetic dimension)

Wicker (1968) conducted an intriguing pair of early studies designed to investigate the “intersensory dimensions in perceptual or cognitive space, *i.e.,* of dimensions which are significantly descriptive of sensory inputs from more than one modality” (Wicker, 1968, p. 178, italics in original). In a first experiment, Wicker’s participants were presented with a range of 13 pure tones (300, 400, 500, 600, or 700 c.p.s.) of varying loudness (53–84 dB); they were also presented with 13 coloured Munsell colour squares (green, red, blue, and yellow) of varying brightness and saturation. The participants had to rate the similarity of all pairs of tones and thereafter to rate the similarity of all possible pairs of colour patches. They also had to rate every individual tone and colour patch in terms of 25 semantic differential adjective scales (cf. Moller et al., 2009), as well as to rate the similarity of the auditory and visual stimuli. In particular, they had to rate the similarity of every tone to every colour using a 9-point scale (Wicker, 1968, p. 180).

Multivariate scaling revealed two orthogonal alignments underlying the intersensory and cognitive space: pitch-brightness and loudness-contrast. The correspondences between these dimensions were established using multidimensional-scaling (MDS), semantic-differential (SD) scaling, and an intersensory transfer of training paradigm. At the same time, however, Wicker’s (1968) results failed to provide evidence of the existence of any crossmodal correspondence between pitch-saturation, loudness-brightness, and loudness-darkness. These findings would therefore appear to suggest that the mapping of colour to sound is likely to have been based on frequency (in terms of the low-high continuum) while ignoring octave repetition.^11^

While it is commonly accepted in the academic literature that it may be possible, and meaningful, to make judgments concerning the perceptual similarity of pairs of stimuli presented within the same sensory modality (Ekman, 1954; Ekman et al., 1964; Marks & Bornstein, 1987; Shepard, 1962, 1974; Tversky, 1977; von Helmholtz, 1878/1971), talking about the perceptual similarity of stimuli presented in different sensory modalities is more controversial, and has attracted a much more diverse range of theoretical responses (e.g., Di Stefano & Spence, 2023; O’Regan, 2011; Spence, 2022). For example, von Helmholtz refuted the very idea that perceptual similarity had any meaning across the senses when he wrote that “the distinctions among sensations which belong to different modalities, such as the differences among blue, warm, sweet, and high-pitched, are so fundamental as to exclude any possible transition from one modality to another and any relationship of greater or less similarity. . . . Comparisons are possible only within each modality” (von Helmholtz, 1878/1971, p. 77; though see Hartshorne, 1934).^12^ Lawrence Marks (2011), by contrast, had the following to say concerning: “Perceptual similarities between and among sensory experiences in different modalities. Much as the color aqua is more similar phenomenologically to cerulean than to pink, the flavour of lime more similar to lemon than to banana, so too are low notes played on a bassoon or an organ more like dark colors such as brown or black than bright colors such as yellow or white, while the higher notes played on clavier or a flute resemble yellow or white more than brown or black” (p. 52). Elsewhere, Marks (1996) suggests that the best we can hope to achieve is what he once called ‘perceptual metaphors’—a term that can perhaps be taken to be synonymous with synaesthetic metaphor.

The above quotes thus demonstrate that eminent psychophysicists have seemingly taken diametrically opposed positions concerning the very possibility of grounding a meaningful translation between the senses on perceptual similarity. At the same time, however, more general concerns can be raised regarding perceptual similarity when the notion is evoked in the cross-sensory context to explain why it is that certain complex stimuli are associated with one another. For example, a funeral march might be associated with a weeping willow as they are both perceived to be sad. In such a case, sadness is the property shared by the paired stimuli. However, the fact that people tend to associate X to A more than to B, for instance, the song “Happy Birthday” to sunflowers more than to weeping willows, does not in-and-of-itself necessarily imply that X is perceptually similar to A. While audiovisual semantic congruency^13^ is established on the basis of the regular co-occurrence of the component auditory and visual stimuli, this does not have any necessary implications for the question of whether the component stimuli are themselves perceived as being in any way perceptually similar (Wegner-Clemens et al., 2022; see also Di Stefano & Spence, 2023).

Furthermore, demonstrating the existence of a statistically significant (or consensual) crossmodal correspondence between stimuli only shows that the pairing was the best of the options that were made available for participants at the time that they were asked (see Spence & Levitan, 2021, on this point). Thus, the mere fact that a robust crossmodal correspondence can be established between two stimuli does not mean that the stimuli are necessarily similar in some/any respect. For example, if English-speaking participants were to be exposed to the word ‘apple’ and to the images of an apple and of a tree, and were asked to match the word with one of the two images, they would undoubtedly match the word to the image of the apple. Clearly, though, in this case, the word ‘apple’ is not more perceptually similar to the apple than it is to the tree. Note that according to embodied theories of language processing (e.g., Barsalou et al., 2012), the exposure to words is thought to activate perceptual representations that are consistent across individuals. However, this does not mean that the words are in any intuitive way similar to the representation they elicit, for instance the word “apple” to the apple, nor that any kind of similarity triggers the perceptual representation, which can probably be more easily explained in term of association.

Another theoretical issue would appear to weaken the idea of perceptual similarity based on shared phenomenological properties even further. The problem here is that a thing might be an ‘A-thing’ with respect to ‘A-ness’, and at the same time a ‘B-thing’ with respect to ‘B-ness’ (see Rodriguez-Pereyra, 2002, for an extensive discussion of this problem). To give an example, to help make this point more concrete, say “redness” is A-ness, while “being a fruit” is B-ness. A strawberry is both an A-thing and a B-thing. So, it is similar to all red things, say cherries and blood, but also to peaches and grapes. This suggests that grounding similarity relationships on shared phenomenological properties will likely make similarity a universal relationship (you will always find an A-ness according to which two different things are similar). Critics point to the fact that any two objects might share at least one phenomenological property and thus, as Goodman (1972) has argued, similarity would simply be a universal relation—namely, everything would be similar to everything else—and therefore claims regarding similarity would become somehow meaningless. Moreover, different properties count differently as far as perceptual similarity is concerned. For example, a strawberry is more similar to a blueberry than to blood, despite both strawberries and blood being red. Thus perceptual similarity would appear to depend on more than just a simple count of shared and unshared perceptual features or attributes; that is, it depends on emergent Gestalt properties (e.g., Palmer, 1989; Pomerantz et al., 1989; Di Stefano and Spence (2023) recently presented an account of perceptual similarity based on a two-dimensional space with associative strength on one axis, and cognitive penetrability on the other).

A further possibility we will investigate is simply that there is no similarity between perceptual dimensions across the senses, but rather that there exist a certain subset of perceptual dimensions that are amodal, or supramodal, meaning (at least according to certain commentators) that the same information is picked-up regardless of the sensory source (Walker-Andrews, 1994). O’Malley (1957, p. 392) talks of this as ‘intersense analogy’,^14^ going on to say that: “In literary discussions, intersense analogy and clinical synaesthesia are seldom distinguished, but it is important to stress their essentially different implications. The principal difference concerns the question of whether or not intersense comparisons or resemblances are accessible to normal, if heightened, experience. For clinical synesthesia, the question of resemblance is incidental; for intersense analogy, it is essential” (O’Malley, 1957, p. 393). Notice how here, O’Malley would appear to be drawing attention to the idiosyncratic nature of the inducer-concurrent mapping in synaesthesia.

### Amodal properties

More than two millennia ago, Aristotle drew attention on the existence of features of the world that can be perceived in their own right by different senses: “For the perception of magnitude, figure, roughness, smoothness, and sharpness and bluntness, in solid bodies, is the common function of all the senses, and if not all, then at least the common function of sight and touch” (*De Sensu et* *Sensibili*, 442b in Aristotle (1908); see also *De anima*, 418a10–11, 19 in Aristotle (1907); see also Paterson, 2021, p. 33; Werner, 1934, p. 202). Based on such an almost synaesthetic view of perception, some researchers have wanted to suggest that amodal sensory dimensions might therefore provide a robust basis for connecting the senses. A number of developmental psychologists have argued that amodal stimulus dimensions provide a fundamental role in terms of helping to scaffold multisensory interactions in human development (e.g., Bahrick, 2010; Bahrick et al., 2004; Bahrick & Pickens, 1994; Gibson, 1969; Lickliter & Bahrick, 2012; Smith, 1987).^15^ Consider here only how people can recognize the same temporal pattern no matter whether the information is provided via hearing, touch, or vision (e.g., Marks, 1987a; see also Frings & Spence, 2010; Marks, 1987b), thus suggesting that such mechanisms that enable the processing of temporal patterns are not modality-specific, and hence that temporal pattern is likely to be an amodal stimulus property. According to Bahrick (2009): “Properties of objects and events such as temporal synchrony, rhythm, tempo, duration, intensity, and co-location are common across auditory, visual, and proprioceptive stimulation” (p. 44; see Grahn, 2012; Huang et al., 2012; McAuley & Henry, 2010). Taken together, such results would appear to support Lewkowicz and Turkewitz’s (1980) early claim that rhythm is an amodal dimension. A similar argument has been made with respect to numerosity (see Gallace et al., 2007). It should, though, be noted that both numerosity and rhythm are linked to the organization of groups of stimuli and are not themselves sensory properties of the stimuli (i.e., in the way that stimulus intensity is, say).

Bahrick (2009) notes that amodal literally means “without” modality. However, she chooses to ignore this use of the term (e.g., as the term is used in the literature on the perceptual completion of occluded stimuli). Instead, the dimensions that Bahrick considers amodal consist of a mixture of those that are amodal by virtue of the fact that different senses sometimes pick up the same sensory information, such as vision and touch providing information about the size and shape of hand-held objects, and those that are amodal in virtue of the fact that the perceptual quality is somehow equivalent across different senses, as in the case of stimulus intensity. Note, though, that there is seemingly no explicit necessity for the relevant unisensory experiences nor for what is being picked-up from different senses to be phenomenally similar (Ernst & Banks, 2002). According to Bahrick (2009), “Amodal information includes changes along three basic parameters of stimulation—time, space, and intensity” (p. 44). Marks et al. (1987) also talk of the “perceptual, cross-modal equivalence with respect to intensity” (p. 5).^16^

One of the earliest studies on amodal dimensions was published by von Hornbostel (1931). Von Hornbostel’s hypothesis was that brightness represented a universal dimension of sensory experience. The small number of participants (*N* = 3) in his study had to match sounds of different pitches to points along a greyscale. They also crossmodally matched scents with grayscale values. Given the apparent transitivity between different crossmodal comparisons, von Hornbostel interpreted his results as demonstrating that the concept of ‘sensory brightness’ must be common to all of the senses. Were this, in fact, to be the case, one could easily imagine how simply matching the brightness of auditory and visual stimuli would provide a means of meaningfully (or consensually) translating between the senses.^17^

Even early researchers were not entirely convinced of the existence of amodal stimulus dimensions (see Cohen, 1934), arguing instead for a relative/relational judgment account (i.e., rather than necessarily a crossmodal perceptual mapping based on amodal properties; cf. Hartshorne, 1934). As Cohen (1934, p. 119) put it, and importantly for our concept of sensory translation (where two different stimuli are necessarily at stake), the stimuli in von Hornbostel’s study were ‘analogous’ rather than ‘identical’. Cohen tried to explain his reasoning as follows: “It would not be unreasonable then to suppose that cross-modality comparison should be based (physiologically, if not introspectively) upon relative positions within different ‘absolute’ scales. According to this view equation with respect to brightness of two experiences of different modalities would involve nothing more than the identity of relative positions upon two wholly independent scales.” According to Marks et al. (1987, p. 34): “As a general rule, psychophysicists who study crossmodal matching have concerned themselves primarily with determining precise quantitative measures of intersensory equivalence; their purpose is usually to test theoretical predictions made from psychophysical functions (which relate judgments of sensory magnitudes to physical intensities) derived for individual continua like loudness and brightness.” In other words, while a robust psychophysics of crossmodal matching is consistent with the existence of an underlying amodal dimension guiding people’s choices, it certainly doesn’t entail it.

Mellers and Birnbaum (1982) describe the distinction thus:

Two prominent theories of cross-modality matching are mapping theory and relation theory (Krantz, 1972; Shepard, 1974). According to mapping theory, psychological values of stimuli from different continua are mapped onto a common scale of sensation and can be compared directly. A cross-modality match is presumed to occur when equal strength sensations are elicited by stimuli on different continua. According to relation theory, relationships (e.g., ratios) between pairs of stimuli from different continua are compared. In physical measurement, a mass in grams cannot be compared with a length in centimeters but ratios of masses can be compared with ratios of length. By analogy, it may be possible to compare the ratio of the heaviness of two weights to the ratio of the loudness of two tones, since the ratios of stimulus pairs are on a common scale. (p. 593)^18^

Later, Mellers and Birnbaum (1982, p. 600) go on to suggest that: “In cross-modality judgments, the scale values are influenced by the stimulus distribution: It appears that subjects compare the relative position of a stimulus in its distribution with the relative position of a stimulus of another modality to its distribution”, going on to suggest that their results were consistent with a psychological relativity theory of crossmodality judgment.

The possibility that amodal concept(s) might exist is apparently linked to the existence of absolute correspondences,^19^ as Smith (1987) observed:

This suggestion of a trend from dichotomous, categorical treatments of continua to more relativistic ones ought not to be confused with the issue of absolute versus relative correspondences across dimensions. The notion of absolute correspondences between dimensions is that particular values on one dimension map onto particular values on another—for example, higher is not like brighter; rather, a specific pitch matches a specific brightness. As Marks et al. point out, though, there is little evidence for such absolute correspondences. (pp. 97–98)

Note here also that pitch-based crossmodal correspondences tend to be relative rather than absolute (see Spence, 2019b).

Marks et al. (1987) conclude that “in some fundamental sense, the similarities between pitch and brightness and between loudness and brightness are personal, internal, and subjective; they reside in perception per se and probably depend on common processes of neural coding” (p. 84). Note the strong claim here, albeit with multiple provisos, that similarity relations are perceptual in nature (see Di Stefano & Spence, 2023, for a discussion of the perceptual/cognitive nature of similarity). Nevertheless, the ‘personal, internal, and subjective’ element did not stop Marks (1987a) from trying to establish a robust psychophysics based on the crossmodal matching of the colour lightness of grey surfaces with the pitch of pure tones. However, the ability to crossmodally match stimuli is presumably possible between any pair of prothetic stimulus dimensions, only a few of which might be argued to pick-up on the same stimulus, or perceptual, property (cf. Cohen, 1934; Mellers & Birnbaum, 1982).

To summarize, beyond shedding light on the way in which stimuli are organized within distinct perceptual dimensions in discrete sensory modalities, the distinction between metathetic and prothetic stimulus dimensions leads to the related distinction between absolute versus relative crossmodal correspondences. The latter distinction is, in turn, instrumental when it comes to assuming the existence of amodal concepts, conceived of as the same physical property (such as shape) being picked up via multiple senses (see Lewkowicz & Turkewitz, 1980). This, it should be noted, is subtly different from von Hornbostel’s (1931) notion of universal dimensions of perceptual experience. The emphasis in the latter case would appear to be on the perceptual experience itself (i.e., what it is like), whereas the emphasis for many of the amodal dimensions that have been proposed has been on the multiple routes to picking-up information about physical properties out there, regardless of the perceptual qualities that may be associated with that information.

In addition to these early objections, a number of additional issues have also been raised in the literature regarding the status of amodal properties (see Spence & Di Stefano, submitted). On the one hand, there would appear to be disagreement about how, exactly, amodal dimensions should be defined. As observed by Johnstone (2021), even Aristotle left it somewhat unclear as to whether these common sensibles should be common to all of the senses or just to two or more of them (see also Bahrick, 2009; Gogate & Bahrick, 1998; Walker-Andrews, 1994). Aligning with most commentators (e.g., Knuuttila, 2008), Johnstone takes Aristotle’s considered view to have been that common sensibles are perceptible in their own right by more than one sensory modality, but need not necessarily be perceptible by all five of the commonly accepted senses. Some commentators have suggested that the same dimension, such as sensory intensity (Lewkowicz & Turkewitz, 1981),^20^ sensory brightness (von Hornbostel, 1931; though see Cohen, 1934), or even sensory ‘thickness’ (Moul, 1930) should be considered as amodal dimensions, given that these perceptual attributes/dimensions can be associated with two or more (and possibly all) of the senses. Others, though, have wondered whether the robust psychophysics (e.g., of transitivity) that is obtained when comparing judgments across various pairs of senses (Ellermeier et al., 2021, on the ratio-based crossmodal matching of visual brightness and sound intensity; cf. Heller, 2021; Luce et al., 2010) might not merely reflect the application of ratio properties within qualitatively distinct unimodal prothetic dimensions (see Cohen, 1934; Root & Ross, 1965; Stevens, 1957, 1966, 1971; Stevens & Guirao, 1963). Of course, if the latter suggestion were to be correct then it should not matter which particular pair of prothetic dimensions are chosen for crossmodal matching/scaling. However, those who support the existence/preferential status of certain specific amodal dimensions of experience would presumably have to predict that crossmodal mapping based on a putatively amodal dimension should be more robust, and perhaps also develop earlier, than those correspondences between two distinct prothetic dimensions.

Another possible approach to amodal dimensions is based on the notion of redundant information. As Gogate and Bahrick (1998) put it: “Amodal information is information which is completely redundant across two or more senses” (p. 99). Here, though, it is important to note that there is virtually never perfect redundancy between the senses, even when multiple senses are potentially capable of picking-up on the same environmental property, such as, for example, size/shape (Spence et al., 2013), the precision/accuracy of different unisensory estimates rarely aligns perfectly (Ernst & Banks, 2002). At the same time, and as has already been mentioned, vision and touch only pick up on the same shape/size information over a very narrow range of stimulus sizes.

Ultimately, it is obviously going to be much easier to translate between the senses if there are amodal (and/or absolute) correspondences rather than if crossmodal matches (correspondences) are relative and/or context-dependent. However, while there is evidence for structural translation (e.g., of temporal patterns) across the senses, other dimensions that have been proposed as amodal are, in fact, based on relative (or relational) judgments instead. Returning to the questions that were raised at the start of this article, it can be seen how the existence of amodal stimulus dimensions should allow for a literal, rather than merely metaphorical, translation of a given property. However, while the temporal structure might allow for the matching of a tactile or visual rhythm with an auditory temporal pattern (and so offers the means of conveying the temporal structure) what cannot so easily be captured is the beat attached to auditory temporal patterns (e.g., Grahn, 2012).

#### Affective (emotionally mediated) correspondences

Reviewing the literature, it is striking how many of those artists who have attempted to search for perceptually meaningful correspondences between colour and music have ended up stressing the emotional, or affective, basis of the crossmodal matches that they have intuited, or managed to document empirically (e.g., Bragdon, 1916, 1918, p. 139; Cutietta & Haggerty, 1987; Zilczer, 1987; see Hartshorne, 1934; Marin et al.,  2012; Spence, 2020a, and Spence & Di Stefano, 2022b, for reviews). In fact, emotional mediation has recently been presented as one of the key factors accounting for a very wide range of different crossmodal correspondences (see Spence, 2020a, for a review).

Support for emotional mediation in the case of audiovisual crossmodal correspondences comes from the results of a study published by Palmer et al. (2013), in which the participants had to associate musical excerpts to colour patches and rate both for their emotional valence (e.g., happy, sad, angry, calm, strong, weak, lively, and dreary). The results highlighted significant correlations between the emotional character of the musical excerpts and those of the colour patches that were chosen to match them (see also Bresin, 2005; Simpson et al., 1956; Whiteford et al., 2018).^21^ Along similar lines, Isbilen and Krumhansl (2016) tested their participants with music excerpts from Bach’s *Well-tempered Clavier* and a sample of saturated colours. Their results confirmed that music–color associations can be accounted for by the correlations between music and emotion, and color and emotion. Interestingly, the experimental sample included synaesthetes and those with absolute pitch, who failed to show any peculiar behaviour despite their unusual/extraordinary abilities.

The evidence suggests that emotional mediation is also relevant for those crossmodal associations involving olfaction (e.g., see Levitan et al., 2015, for odour–music associations; Schifferstein & Tanudjaja, 2004; and Gilbert et al., 2016, for colour-taste/smell associations; Di Stefano et al., 2022a). Winter’s (2016b) findings might also be taken to indirectly support such a central role of emotion, explaining why it is that taste (gustation), in particular, is a common source domain in most of the crossmodal correspondences, as taste and smell are simply more strongly emotionally valenced (see Levinson & Majid, 2014; Winter, 2016a).

Based on Spence’s (2020a) review, it would seem that emotion offers a crucial explanatory concept underpinning the majority of audiovisual associations (see Hartshorne, 1934, for a similar position). That being said, several relevant questions arise here. First, one reviewer of this manuscript wondered why colour should be mapped to emotion in the first place (D’Andrade & Egan, 1974; Jonauskaite et al., 2020). In this case, the answer may well relate to the fact that exposure to different colours has been documented to directly affect people’s emotions (Jonauskaite et al., 2019). So, for example, exposure to a Baker-Miller Pink environment may help to calm people (Schauss, 1981, 1985; though see Genschow et al., 2015). Relatedly, it is legitimate to ask whether emotional associations can also be invoked to explain mappings between pitch, brightness, and loudness. This would seem unlikely, but returning to the debate between von Hornbostel, Cohen, and Hartshorne discussed earlier, this might better be considered as a relational (or relative) correspondence, i.e., a kind of analogical mapping (Ravignani & Sonnweber, 2017). Readers wanting to know more about the importance of emotional mediation to explaining many audiovisual crossmodal correspondences are directed to the reviews by Spence and Di Stefano (2022a, 2022b), where the emotional mediation account of audiovisual correspondences is discussed in much more detail.

## Problems for any attempt to translate directly, or ‘literally’, between the senses

There are a number of further potential problems for anyone wanting to translate between the senses (in particular, between individual sensory impressions); these relate to attempts to extend from the crossmodal pairing of individual auditory and visual stimuli to matches between more complex combinations of sensory stimuli (Marks & Bornstein, 1987).

### Intramodal versus crossmodal grouping

Intramodal perceptual grouping (defined in terms of the Gestalt grouping principles; see Wagemans, 2015) tends to be much stronger than crossmodal perceptual grouping (see Spence, 2015, for a review). As such, any association, or correspondence that might be established between a specific colour and a particular sound (Sebba, 1991) is likely to be overridden by the emerging intramodal perceptual grouping that will likely take precedence as soon as more than one stimulus is presented in either modality (cf. Bhattacharya & Lindsen, 2016; Collier & Hubbard, 2001; Cuddy, 1985; Cutietta & Haggerty, 1987; Galeyev, 1976; Platt et al., 1990; Woods et al., 2016; Woods & Spence, 2016). Note that this observation can be seen as following naturally out of the fact that perceptual similarity can be more easily understood when occurring within, but not between the senses, given that ‘grouping by similarity’ is one of the central Gestalt grouping principles. Similarly, consider here only how a given sequence of musical notes may be associated with positive emotion if the sequence is arranged as an ascending pitch series, but the same sounds when organized as a descending sequence, is associated with negative emotion instead. In all these cases, the meaning is linked to structure of elements or emergent property (see also Cuddy, 1985).

As soon as one starts to look at the correspondence between works of art (e.g., when looking for correspondences between pieces of music and paintings), then the influence of cross-media artistic styles starts to become increasingly relevant (e.g., Arnheim, 1974, 1986; Dailey et al., 1997). In such cases, the audiovisual correspondence may be based on the higher-level structural processes due to language, culture, abstract symbolization, learning, rather than any particular association that may exist between the individual component stimuli (e.g., colours/shapes or musical notes; e.g., Actis-Grasso et al., 2017; Adams, 1995; Albertazzi et al., 2015, 2020; Duthie, 2013; Duthie & Duthie, 2015). Some have even referred to aesthetic correspondences between the arts (Schueller, 1953).^22^ Notice how, in all such cases, it’s the Gestalt organization (or artistic style) that likely dictates the crossmodal matches that are deemed most appropriate. According to O’Malley (1957):

Interrelation of the arts, taken somewhat for granted in most discussions of aesthetic movements or tempers, tend to resist exact definition because they depend more on vague complex similarities in the general aims and expressive ideals of artistic generations than on easily recognizable resemblances among elements of the several arts. Even in attempts to compare such elements (as in equating figurative design with melody, or coloring with harmony), the analogy refers essentially to broad similarities in formal functions, not to specific resemblances between impressions of different senses. Care should be taken, therefore, to distinguish between the correspondence of the arts and intersense analogy. (p. 402).

Similar observations also hold for the combination of soundtracks with movies, with studies showing that composer-intended music-film combinations tend to be selected by participants as providing the best fit (Lipscomb & Kendall, 1994). However, in such cases, it may be more of an affective match (cf. de Staël, 1869, pp. 485–486; O’Malley, 1957, p. 403). Furthermore, the apparent synchronization of the component stimuli may also play an important role as well (see Daurer, 2010; Muller, 2010). At the same time, however, Stechow (1953, p. 324) notes how “vague associations between music and architecture are not very rare. In a sense, Moussorgsky’s Gate of Kiev can be considered as such rather than as a translation into music of Victor Hartmann’s drawing (Frankenstein, 1939).” Any correspondence that is experienced in the latter case might well be considered to be based of transfer from the temporal aspects of (auditory) to the spatial (visual) domain (Julesz & Hirsch, 1972).

### Direct association versus perceptual inference

Intriguing work from Schloss et al. (2018) has highlighted the fact that people’s interpretation of the ‘meaning’ of a given colour may sometimes be determined not by the strength of any direct crossmodal mapping but rather by whatever other stimuli happen to be in the comparison set. As such, colour mappings (and presumably any other kind of crossmodal mapping) may sometimes be inferred rather than necessarily signalling the strongest possible association between the component stimuli that are available for comparison (Mukherjee et al., 2022). As such, in judging the efficacy of any attempt to translate between the senses, it may be important for the interpreter (that is, the person trying to make sense of the stimuli) to know the intention of whoever came up with the translation scheme (i.e., the code mapping the one sensory stimulus to another, and/or the range of stimuli) in the first place, and also for there to assume a communicative, or signalling, function behind the selection, or choice, of colours. This notion is referred to as ‘semantic discriminability theory’ by Mukherjee and colleagues. There may also be a link to the literature on colour-in-context theory here (Elliott & Meier, 2012, 2014). According to the latter account, the meaning that is attached to a given colour depends on the context in which it is presented. Think only of how red primes temperature (i.e., hot) in the context of taps (where the contrast is with blue) whereas red primes stop/danger when in the context of traffic (and when the comparison may be green; e.g., in traffic lights). So, even if the comparison set does not provide a context as such (e.g., in the sense of ‘colour-in-context’ theory), nevertheless, at least according to semantic discriminability theory, participants’ choices might be constrained by the range of stimuli people are given to respond with. Once again, note how these concerns argue against an absolute mapping between the senses, and thus an easy translation of one sense to another.

### Transduction, mimesis, analogy, and parallelism

We conclude by briefly mentioning a few other notions that have not been touched on in this review, but might be considered relevant when attempting to explain sensory translation. First, transduction (Helmreich, 2015a, b; Lick, 2022; Newfield, 2017), which can be defined as the process taking place when many sensors in the body convert physical signals from the environment into encoded neural signals sent to the central nervous system (Schacter et al., 2010). It should, though, be noted that transduction, strictly defined, is a biologically-determined process as there is not really any choice about the conversion of sensory input in a particular pattern of neural signals (though see also Culache, 2015a, 2015b). Taking a broader perspective, Lick (2022, p. 6) suggests that: “Transductions have been studied within different fields and research areas, for example translation studies, built environment, and education. In general, when adopting a multimodal discursive approach, the term “transduction” (Kress, 2010), also referred to as “resemiotization” (Iedema, 2003), pertains to situations where meaning is shifted from one mode to another, such as written information in a report, which is visualized in a diagram (writing vs. picture). Whereas this transduction uses modes from the same modality (visual), transductions may also be performed by changing from one modality to another, like a presenter’s speech which is simultaneously projected on the wall behind them (auditory vs. visual; Jewitt et al., 2016; Kress, 2010). It must be mentioned that in any transduction the overall meaning of the multimodal text needs to be maintained to ensure the intended interpretation processes (Culache, 2015a, b).”

The notion of mimesis has been evoked by Connor (2004) to account for the relations between sound and touch as follows: “The relations between sound and touch … tend to be mimetic: Touch accompanies, mimics, performs sound rather than translating … it” (p. 154; cf. Taussig, 1993, on the notion of mimesis). By contrast, sensory translation occurs primarily, and more properly, between sound and sight, with the information provided by sight being the transformation of the one obtained auditorily. It should also be stressed that Piesse (1867, 1891), the chemist and perfumer, would appear to have been more interested in drawing analogies between the ways in which elements within the auditory and olfactory modalities could be combined harmoniously (see also Cooke & Myin, 2011), rather than necessarily on establishing any direct crossmodal perceptual match (or assert any form of perceptual similarity) between individual auditory and olfactory stimuli. Several commentators have thus referred to the notion of colour-tone analogies (Gombrich, 1979; Jewanski, 2010b; Jewanski & Naumann, 2010; see also O’Malley, 1957).^23^ Some years ago now, Stechow (1953) had already highlighted an important distinction between different kinds of relation: “*translations* from the visual arts into music and *parallelisms* between the visual arts and music” (p. 324, italics in original). Later, he observed that “it would seem to me that comparability of structure reveals a more ‘real’ relationship between such works of art than a mere affinity of ‘mood’ or ‘texture’ could suggest” (Stechow, 1953, p. 325). The latter comment presumably emphasizing the structural rather than the affective nature of correspondences.

## Conclusions

As this narrative historical review has hopefully made clear, people have been interested in translating between the senses for millennia (see also Spence & Di Stefano, 2022a, b, for reviews). Beyond philosophers and artists, researchers have also been attracted by sensory translation in their attempt to explain its underlying psychological mechanisms. Several perceptual phenomena have been evoked, such as synaesthesia and crossmodal correspondences. And while traditional synaesthetic and structural mapping approaches have largely failed to explain sensory translation (at least they have failed in the sense of not providing a broadly consensual crossmodal mapping; see Jewanski, 2010a), crossmodal correspondences would appear to offer an alternative way of thinking about the translation between the senses (see Arnheim, 1986, for a broadly similar conclusion).

However, the majority of the evidence that has been published to date suggests that an approach based on affective, or emotionally-mediated, crossmodal mappings is more likely to work than attempts to search for perceptual (possibly amodal) correspondences that are based on putative crossmodal perceptual similarity instead (cf. Hartshorne, 1934; Spence, 2020a; von Helmholtz, 1878/1971; though see also Marks, 2011). Ultimately, therefore, it can be argued that the best that one can hope for as far as matching, or translating between, the senses is to use emotionally mediated correspondences (e.g., Cunningham & Weinel, 2016; Hauck et al., 2022; Spence, 2020a; Spence & Di Stefano, 2022a, for a review). Evidence showing that basic emotions are recognized in musical stimuli across cultures (Fritz et al., 2009) would at least partially support the alleged universality of some elements of emotion-mediated translation from audition and vision. That being said, though, there are presumably only a relatively limited range of distinct emotions (or emotional states) to play with, thus likely limiting the range of possible translations.

Returning, then, to the three key research questions that were outlined in the Introduction: (1) How the topic of sensory translation is related to synaesthesia, multisensory integration and crossmodal associations? (2) Are there common processing mechanisms across the senses that guarantee for sensory translation or, rather, is mapping among the senses based on allegedly universal stimulus dimensions (e.g., amodal)? (3) Is the term ‘translation’ in the context of sensory mapping used metaphorically or literally? In answer to the first question, the topic of sensory translation is related to the topic of synaesthesia because the vivid concurrents experienced by synaesthetes have often been considered as providing guidelines for appropriate translation, especially in the field of arts (seemingly neglecting the fact that synaesthesia is defined by the idiosyncrasy of the inducer-concurrent relations). Meanwhile, the link between sensory translation, multisensory integration, and crossmodal associations can be seen in terms of the emerging literature demonstrating that crossmodal correspondences both modulate multisensory integration and also provide a more consensual guide to translating between the senses (see Pinardi et al., 2023). Regarding the second question, the reviewed literature seems insufficient to support the existence of processing mechanisms that guarantee for sensory translation across the senses. Rather, it would seem to suggest that the only common processing mechanisms across the senses may relate to magnitude (e.g., Pinel et al. 2004; Walsh, 2003; though see also Ronga et al., 2012), though further, and more solid, evidence is required in this direction. At the same time, although universal stimulus dimensions (e.g., amodal or intersensory) have been proposed by a number of researchers over the years, convincing empirical support for their existence has not been forthcoming. Finally, the answers to the first two question provide indications to address the third one. Observing that, with the possible exception of some theoretically extreme positions (e.g., Paradis & Eeg-Okofsson, 2013; Rakova, 2003), the term ‘translation’ when used in the context of sensory mapping would appear to be used metaphorically rather than literally, thus indicates that the relationship between the sensory impressions that are being ‘translated’ is based on a merely perceptual basis, being it semantic, emotional or cognitive.

While the discussion in the latter parts of this review has focused specifically on attempts to translate between auditory and visual stimuli, it is worth noting that a growing number of crossmodal correspondences have now been documented between the other senses as well (e.g., olfaction, gustation, and touch; e.g., Belkin et al., 1997; Crisinel & Spence, 2012b; Di Stefano et al., 2022a; Gilbert et al., 1996, 2016; Kemp & Gilbert, 1997; Motoki et al., 2020;  Motoki et al., 2022; Piesse, 1867, 1891; Raevskiy et al., 2022; Spence, 2020c; Spence et al., 2015; Watson & Gunter, 2017). However, here again, in those cases where a specific source object cannot be identified,^24^ emotional mediation appears to provide the most parsimonious explanation for the various mappings (e.g., between hue and olfaction, or between hue and colour) that have been documented to date (see Gilbert et al., 2016; Schifferstein & Tanudjaja, 2004).

### Directions for future research

People appear to show broad agreement regarding matching in the cases of the crossmodal correspondences, such as those correspondences that have been documented between auditory and visual stimuli. It has also been suggested that they also have a ‘feeling of knowing’ what the consensual (i.e., consensual in the sense of commonly agreed, given that there is no objectively correct answer) answer is likely to be (Koriat, 1975, 1976, 2008, 2011; Rader & Tellegen, 1987).^25^ In the future, it will be interesting to further study consensuality across a given population as well as its consistency within an individual over time, as these may both be considered to provide measures of the strength of such correspondences. Further research is also needed in order to explain why it is that certain correspondences appear to be stronger, or more robust, than others (cf. Parise, 2016). While as yet there has been little research on such issues, it appears that crossmodal correspondences tend to be fairly consistent (or stable) over time (e.g., Belkin et al., 1997; O’Mahony, 1983). It is interesting to consider what the link might be between the strength/consensuality of crossmodal correspondences and the directionality of synaesthetic metaphor. This is undoubtedly an area where additional research is very much needed.

Looking to the future, protocols investigating sensory substitution might shed light on the way the sensory system can manage the transformation of information from one sensory modality into another (see Pinardi et al., 2023). It will also be interesting to keep an eye on the machine learning literature in order to see whether such big data approaches are capable of turning up any crossmodal matches that work better (in the sense of being more consensual) than those that have been uncovered to date that been based on intuition, synaesthesia, structural alignment, or, on occasion, experimentation (Murari et al., 2020; see also Conway & Christiansen, 2006).

## Footnotes

1 See Castel (1725, 1735, 1740, 1751, 1926a, b) for earlier accounts of an ocular harpsichord (Franssen, 1991; Hankins, 1994; Schier, 1941). According to Evans (1973, p. 190), in the late 1500s, the learned could even hope to perfect a ‘perspective lute’ that linked colours to music tones in synaesthetic union. There was also Athanasius Kircher’s (1602–1680) suggestion that each musical sound corresponded systematically and objectively to a certain colour (Cytowic, 1993; Kircher, 1650).

2 It might be interesting to note that, at that time, even most traditional instruments, such as the piano or the strings, had not reached their actual contemporary form yet.

3 Think here also of ‘herbal capriccios’ from Aldous Huxley’s (1932) novel *Brave New World* (cf. Huysmans, 1884/1926).

4 See https://www.changheelee.com/essence-in-space.html

5 Here, and throughout the paper, the adjective ‘unidirectional’ is attributed to a perceptual or sensory phenomenon that occurs from one sensory modality to another but not in the reverse direction. By contrast, the term ‘bidirectional’ refers to those perceptual phenomena that can be experienced both from one sense to another and vice versa.

6 Just take the following from British wine writer Hugh Johnson (2005): “I have tasted first-attempt Chardonnays that were like Dizzy Gillespie’s solos: all over the place. And the colour of his trumpet, too. On the other hand a Stony Hill Chardonnay recently had the subtle harmonies and lilting vitality of Bix Beiderbecke. Robert Mondavi’s Reserve Cabernets are Duke Ellington numbers: massed talent in full cry. Benny Goodman is a Riesling from Joseph Phelps, Louis Martini’s wines have the charm and good manners of Glenn Miller. Joe Heitz, though, is surely Armstrong at the Sunset Café; virtuoso, perverse and glorious” (p. 253).

7 Waterworth (1997) introduces the term ‘synaesthetic media’, writing that: “The author posits that computer-based multimedia should be considered ‘synaesthetic media’ due to their capacity to translate between modes of sensory perception” (p. 327).

8 Indeed, it has been suggested that the more successful literary metaphors may be those that are more immediate, with Downey (1912) suggesting, “It may be stated as a principle of interpretation that if a given transfer of sense-qualities is found pleasing only when the reader makes the suggested translation easily and spontaneously, there is some evidence that the writer himself used the analogy spontaneously rather than reflectively unless the expression be purely conventional” (p. 497). According to O’Malley (1957), “literatery synaesthesia may imply a sort of introspection into basic processes of experience, a mirroring or echoing of the mind and senses to themselves in the act of apprehending phenomena” (p. 392). To be clear, synaesthetic metaphors are linguistic expressions in which a concept from one sensory domain is described in terms of another sensory domain (O’Malley, 1957, p. 391).

9 According to Paradis and Eeg-Olofsson (2013, p. 37): “We call into question the metaphorical approach to words denoting sensory perceptions offered by Shen (1997) and Shen and Gadir (2009) which states that conceptual mappings from lower and more accessible concepts onto higher and less accessible are more natural than the other way round. They define conceptual accessibility through the directness of contact between the type of sensory perception and the object of experience. This explanation is dubious in many ways. Firstly, the notions of lower and higher modalities are not defined or agreed upon in the literature. In what sense is touch more accessible than smell and taste and why is vision not the most accessible modality?”

10 These, note, linking to the semantic differential approach (Osgood et al., 1957).

11 Cf. Cytowic and Wood (1982), and Ries (1969), for a couple of other examples of participants being asked to pick colours to match a range of tones.

12 Or, as Dr. S. Johnson (1751) put it: “Sound can resemble nothing but sound.”

13 The term ‘semantic congruency’ is somewhat confusingly used in different ways in at least two different literatures. Here, the term is used to refer to those situations where the different sensory properties of a source object, such as the sight and sound of a dog, are combined in semantic representations, as a result of prior co-occurrence (see Chen & Spence, 2011; Hsiao et al., 2012). Note that semantic congruency has no necessary relation to perceptual congruency, the latter being based on perceptual similarity. Consider here only the fact that while the image of the dog and the sound of its bark are semantically connected, they are not in any sense perceptually similar (see also Spence, 2022, on this distinction as it pertains to the chemical senses).

14 O’Malley (1957) writes that “intersense analogy stands for the supposed comparability or resemblance of qualities which are perceived by different senses and may refer to single, tentative parallels like Aristotle's or to detailed and systematic comparisons of the data of two or more sensory modes” (p. 392).

15 For example, just take the following suggestion from the developmental psychologist Linda Smith (1987) that “we have a lot to gain if we can get beyond mapping specific dimensions one to another and instead delineate the amodal dimensions” (p. 96). Clark (1970), meanwhile, argues that polar dimensions are fundamental to children’s development of relational concepts.

16 Paradis and Eeg-Olofsson (2013) write that “as pointed out by Rakova (2003, p. 49), similarities due to perceptual equivalence, rather than conceptual analogies have been extensively studied. Those studies confirm that the perception of cross-modal similarities is universal, systematic and present in early childhood. What these results suggest then is that transitions across sensory domains in human language and understanding are monosemous and syncretic rather than metaphorical and polysemous. Rakova (2003, p. 142) claims that one may think of concepts such as BRIGHT, SHARP, and COLD as primitive concepts spanning all domains of sensory experience. They may be thought of as neural configurations responsive to certain stimuli” (p. 37).

17 On sight-sound translation, von Hornbostel observed: “Strange! It is just where eye and ear differ, that their connection has been sought. For colour, like pitch, changes with the wavelength: a fact which misled the physicists. (In reality there is a difference at this very point: brightness in hearing depends on frequency, while brightness in sight depends on amplitude.)” (Von Hornbostel, 1938, p. 212).

18 Mellers and Birnbaum’s (1982) talk of ‘different continua’ presumably supports the view of modality-specific (though comparable) perceptual continua rather than supporting an amodal account.

19 The notion of an absolute correspondence, implying a unique and direct mapping between two stimuli presented in different sensory modalities can be contrasted with relative correspondences, where the crossmodal mapping is not fixed but depends on the comparison set.

20 So, what dimensions count as amodal? According to Lewkowicz and Turkewitz (1980): “Intensity, rate, duration, spatial location, spatial extent, rhythm, and shape all represent amodal features of the world that can be specified in more than one modality. They stand in distinction to such modality specific features of stimulation as redness, sweetness, and pitch” (p. 597). Meanwhile, according to Walker-Andrews (1994): “Amodal properties (size, texture, flexibility, duration, and intensity) that may be picked up by any modality” (p. 42). Von Hornbostel (1931) writes of intensity as a universal dimension of sensory experience (see also Hayek, 1952, p. 21).

21 Bear in mind here also that people often associate emotions with individual sensory stimuli, such as colours, tastes, and shapes (Wang & Zhang, 2015; Zhou & Tse, 2020).

22 The perceived structure of abstract paintings has been shown to be influenced by the structure of music listened to on initial viewing (Lindner & Hynan, 1987). By perceived structure, Linder and Hynan (1987) have in mind whether the music is more or less organized (they contrast highly unstructured avant-garde music vs. highly structured minimalistic music, in particular the first 8 minutes of Bulent Arel’s “Stereo Electronic Music #1” vs. Philip Glass’s “Contrary Motion”). Those participants who heard the minimalistic music later rated the paintings as controlled, ordered, and unified, whereas those who hears the avant-garde music rated the paintings as more accidental, chaotic, and fragmented (cf. Parrott, 1982; Wehner, 1966).

23 O’Malley (1957) ponders: “But how could the spectrum-octave parallel ever have been considered significant for the supposed perceptibility of color–sound analogies? To take it that way was to confuse the physical and psychological, as Rousseau and others in the eighteenth century pointed out” (p. 401).

24 For example, when yellow is picked as the corresponding colour for the smell of citrus because both are linked to the same source object, a lemon (see Spence, 2020d).

25 This is distinct from what is seen in the case of synaesthesia.
